# Solitary fibular metastasis from lung adenocarcinoma with gene mutation: a case report

**DOI:** 10.3389/fonc.2025.1490271

**Published:** 2025-01-20

**Authors:** Jiechen Chen, Ahmad Alkhatatbeh, Yinbing Lin, Xiaolin Lu, Bin Chen, Hongjiang Chen, Xiaohui Lu, Hansheng Wu, Jun Hu

**Affiliations:** ^1^ Department of Orthopedics Surgery, The First Affiliated Hospital of Shantou University Medical College, Shantou, Guangdong, China; ^2^ Department of Radiation Oncology, Shantou University Medical College Cancer Hospital, Shantou, Guangdong, China; ^3^ Department of Thoracic Surgery, The First Affiliated Hospital of Shantou University Medical College, Shantou, Guangdong, China

**Keywords:** solitary fibular metastasis, NSCLC, Osimertinib, case report, multidisciplinary

## Abstract

Solitary fibular metastasis from lung adenocarcinoma is exceedingly rare, with only five documented cases. This report presents a 40-year-old non-smoking Asian male who initially presented with painful swelling in the right knee. Imaging revealed bony destruction of the right fibular head, and further investigations with chest CT, PET/C, pathologic biopsy and genetic testing identified a primary lung adenocarcinoma with EGFR exon 19 deletion mutation. The patient was treated with Osimertinib, resulting in significant tumor reduction. This was followed by thoracoscopic lobectomy and systematic lymph node dissection, and local radiotherapy for the fibular metastasis. The patient experienced complete pain relief and showed no recurrence or metastasis over a 26-month follow-up. This case highlights the diagnostic challenge posed by atypical presentations of metastasis and underscores the importance of comprehensive evaluation, adherence to treatment guidelines, and a multidisciplinary approach in managing oncological patients. The successful outcome in this young patient emphasizes the effectiveness of personalized treatment strategies in optimizing patient prognosis, offering valuable insights for similar clinical scenarios.

## Introduction

Based on updated estimates from the International Agency for Research on Cancer (IARC), global cancer statistics for 2022 indicate that lung cancer was the most commonly diagnosed cancer worldwide, accounting for nearly 2.5 million new cases. (1) This represents approximately one in eight cancers globally, or 12.4% of all cancer cases that year. (1) Bone metastasis is a common occurrence in lung cancer, with an incidence rate ranging from 30% to 40%. (2) Predominantly, metastases favor the spine and the proximal regions of long bones, comprising 50% of occurrences in the spine, 25% in the femur, and 12% in the ribs and sternum, while metastases to other bone sites are rare. (2) Notably, distal metastases beyond the knee and elbow are infrequent, and solitary metastasis to the fibula is exceedingly rare. Only five documented cases of fibular metastasis from lung cancer exist in the medical literature, (3–7) with the present case being the sole instance identified as adenocarcinoma with genetic mutation. Here, we present a case characterized by knee joint pain, ultimately confirmed through comprehensive investigations to be solitary fibular metastasis from lung adenocarcinoma with EGFR exon 19 deletion mutation. The patient was treated with Osimertinib, a third-generation epidermal growth factor receptor (EGFR) tyrosine kinase inhibitor (TKI), as well as definitive local therapy for primary and metastatic sites, resulting in significant tumor reduction.

## Case report

**Figure 1:**
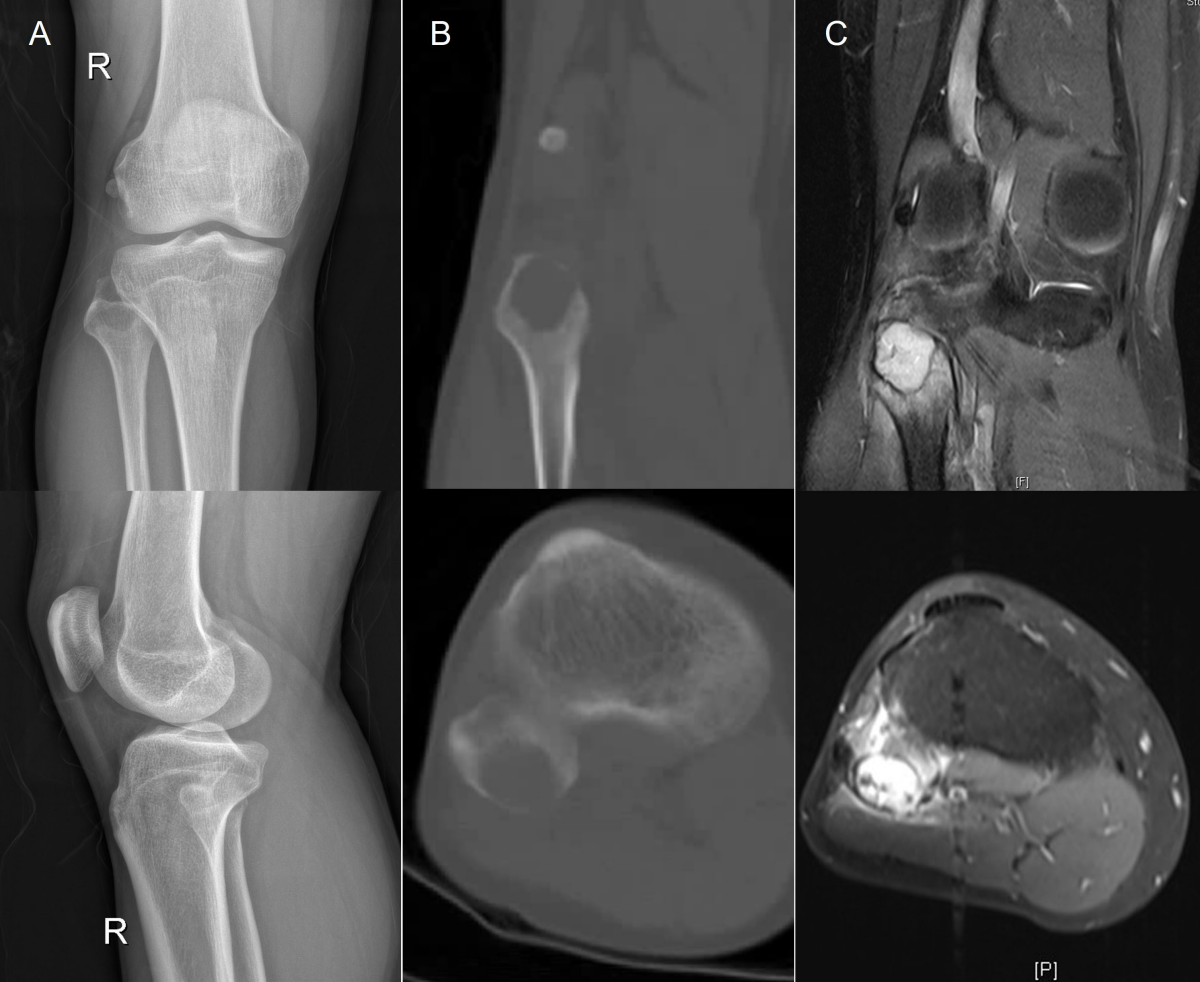
**(A)** X-ray of the right knee joint revealed a rounded, expansive bony destruction of the right fibular head, measuring (23mm×27mm). **(B, C)** CT scan and MRI revealed bony destruction of the right fibular head accompanied by a posterior soft tissue mass, measuring (16mm×24mm), suggestive of a neoplastic lesion. CT, computed tomography; MRI, magnetic resonance imaging.

A 40-year-old Asian male, non-smoker, presented to our hospital on February 6^th^, 2022, with a one-week history of painful swelling and discomfort in the lateral right knee. The patient reported a right proximal calf trauma occurring over three months ago. Physical examination revealed painful claudication of the right lower limb and tenderness over the right fibular head. X-ray of the right knee joint revealed a rounded expansive bony destruction of the right fibular head (**Figure 1A**), CT scan (**Figure 1B**), and MRI (**Figure 1C**) of the right knee joint depicted bony destruction of the right fibular head accompanied by a posterior soft tissue mass, suggestive of a neoplastic lesion.

**Figure 2:**
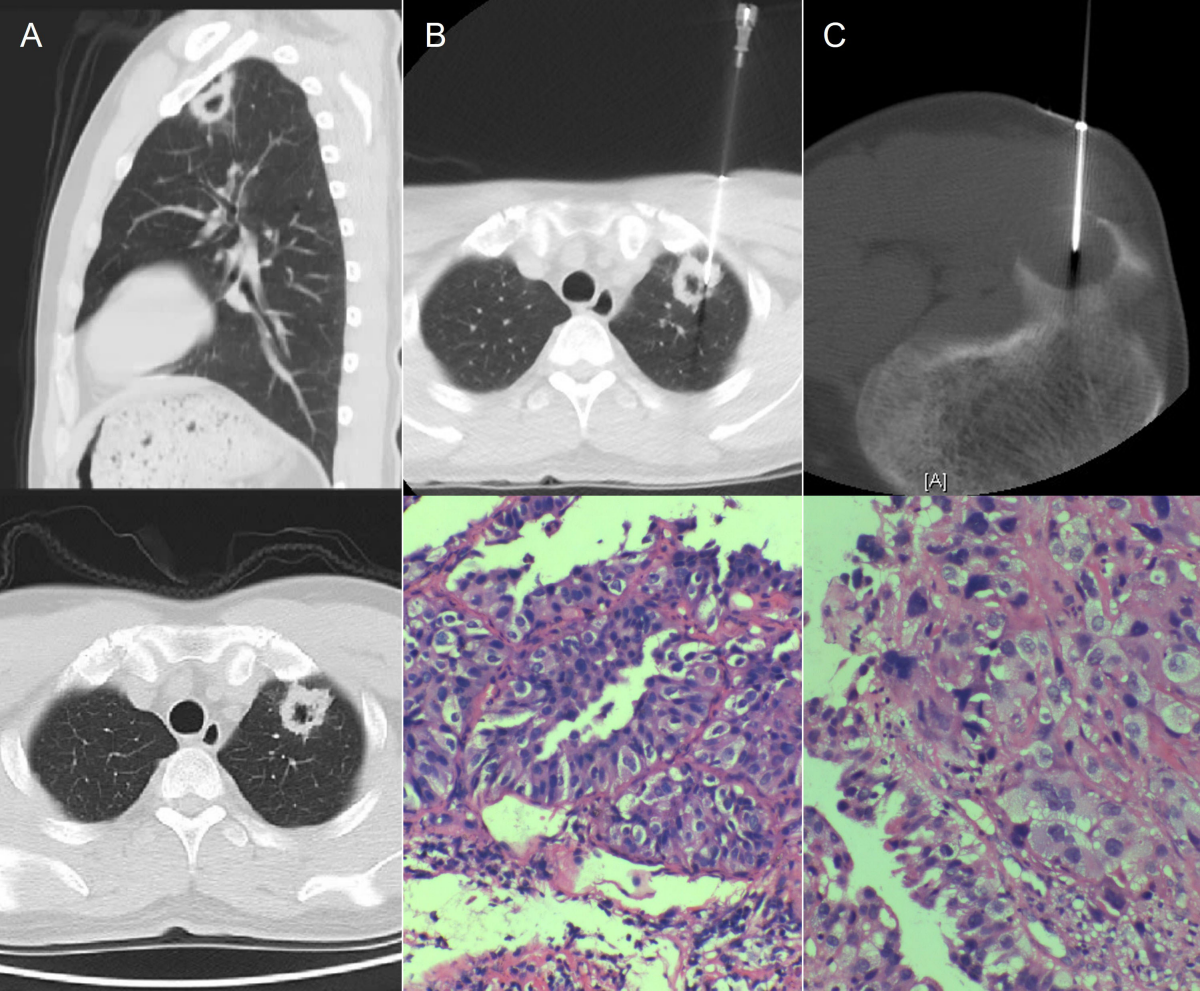
**(A)** Contrast-enhanced CT scan revealed an irregular mass measuring (28mm×26mm×23mm) in the posterior apical segment of the left upper lobe of the lung. CT, computed tomography. **(B)** CT-guided puncture and pathologic biopsy of the right lung nodule suggested primary adenocarcinoma of the left lung. CT, computed tomography. **(C)** CT-guided puncture and pathologic biopsy of the right fibular head mass suggested metastatic adenocarcinoma of the right fibula, possibly originating from the lung. CT, computed tomography.

To ascertain the potential primary site of the neoplasm, chest CT was performed, suggesting an irregular mass, measuring (28mm×26mm×23mm) in the posterior apical segment of the left upper lobe of the lung, suggestive of peripheral lung cancer (**Figure 2A**), and PET/CT suggested a peripheral lung cancer in the apical segment of the right upper lobe of the lung (SUVmax11.3) and metastatic tumor in the right fibular head (SUVmax20.5), no significant abnormal metabolic signs were observed in the brain parenchyma.

Pathologic biopsy and immunohistochemistry of the right lung nodule and right fibular head mass suggested primary adenocarcinoma of the left lung and metastatic adenocarcinoma of the right fibula, possibly originating from the lung (**Figures 2B, C**). This was diagnosed as Stage IVB (cT1cN1M1b). Genetic testing using second-generation sequencing revealed an EGFR exon 19 deletion mutation in tissue biopsy from the right lung mass.

**Figure 3:**
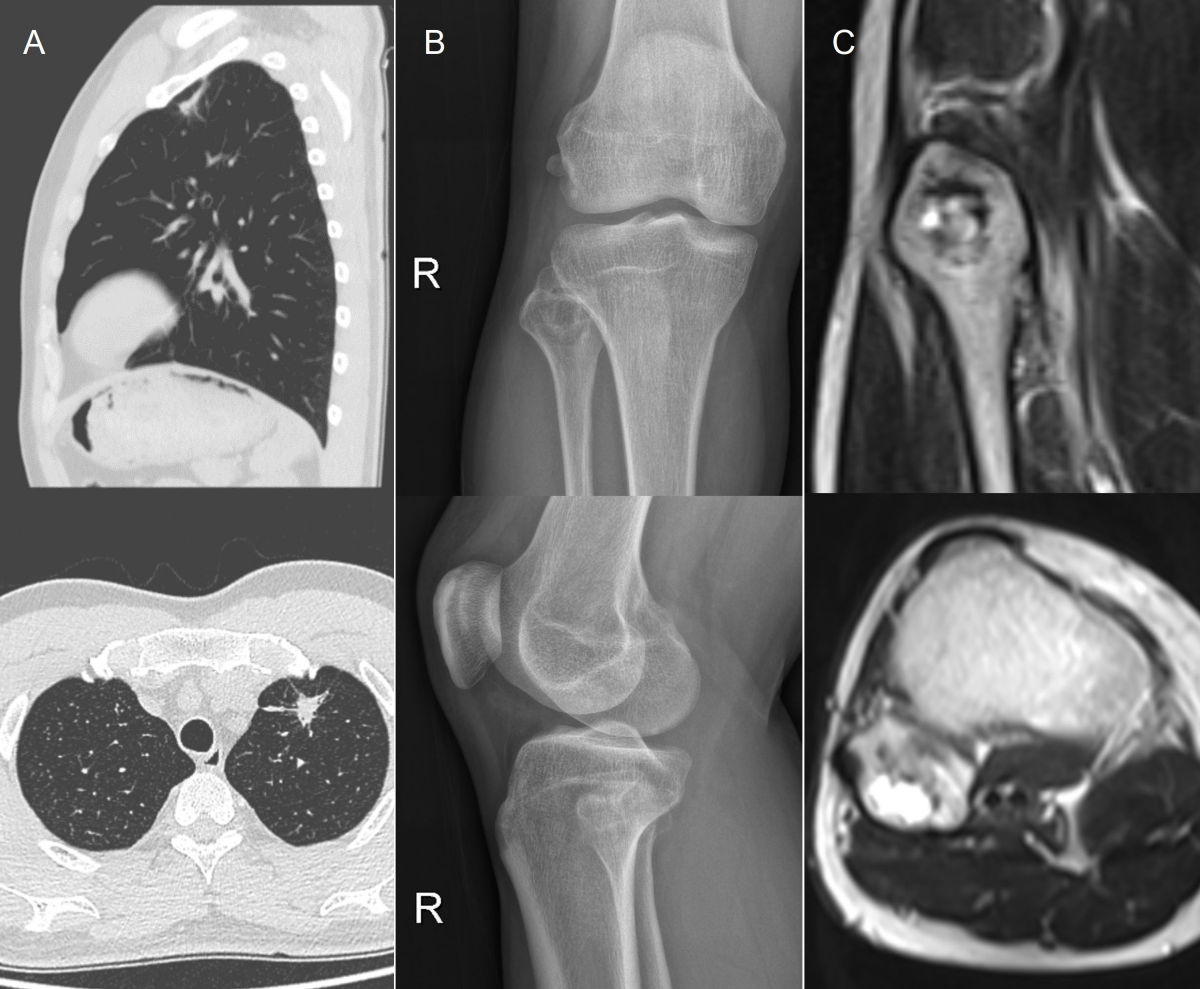
**(A)** Contrast-enhanced CT after Osimertinib therapy revealed a reduction in the size of the lung nodule, measuring (18mm×13mm×9mm). CT, computed tomography. **(B, C)** Post-radiotherapy follow-up X-ray and MRI indicated a decrease in the size of the fibular metastasis lesion, measuring (15mm×17mm). MRI, magnetic resonance imaging.

On April 1^st^, 2022, the patient initiated Osimertinib therapy at a daily dosage of 80mg. Chest CT on June 12th, 2022, revealed a reduction in size of the lung nodule, measuring (18mmX13mmX9mm) (**Figure 3A**), the metabolic activity of the lung cancer (SUVmax2.4) and the lesion at the right fibular head (SUVmax2.6) exhibited significant reduction on PET/CT imaging compared to the previous examination.

On June 15^th^, 2022, a thoracoscopic lobectomy with systematic lymph node dissection was performed on the upper lobe of the left lung. the postoperative diagnosis confirmed Stage IVB (ypT2aN0cM1b). The pathological result reveals pathological tumour regression grade 2 (TRG2, rare residual cancer). On July 26^th^, 2022, the patient underwent local radiotherapy for fibular metastasis. Follow-up examinations post-radiotherapy, including X-ray and MRI, indicated a decrease in the size of the lesion, measuring (15mm×17mm) (**Figures 3B, C**), with the patient experiencing complete pain relief. The RECIST evaluation indicates a Partial Response (PR). The patient has continued Osimertinib treatment following surgery and radiotherapy, with no progression observed to date. Subsequent monitoring, conducted over a period of approximately 34 months, in accordance with NCCN guidelines, revealed no recurrence or metastasis. The patient has generally adhered to medical advice, returning for regular follow-up visits approximately every 3 months.

## Discussion

This case report represents the most comprehensive diagnosis, treatment, and follow-up among existing reports on fibular metastases originating from lung cancer. Additionally, it is noteworthy that among the five existing case reports of fibular metastases from lung cancer, (3–7) this case is particularly significant. It involves the youngest patient among the previously reported five cases at only 40 years old with lung adenocarcinoma, and it is the first case among these with EGFR exon 19 deletion mutation. Furthermore, the initial presentation of pain in the right knee posed a challenge to diagnosis, serving as a valuable reference for similar clinical scenarios.

The diagnosis and management of this case have yielded several key insights which could lead us to an initial approach suggesting upon encountering a patient with a bone mass, comprehensive evaluation including lung CT scans and other relevant tests is imperative to exclude metastasis. This approach prevents diagnostic oversight and ensures timely initiation of treatment. Furthermore, Adherence to treatment guidelines significantly enhances patient prognosis. As per the 2024 National Comprehensive Cancer Network^©^ (NCCN^©^) guidelines, patients with limited-site oligometastatic disease and good performance status (PS) may benefit from aggressive local treatment of both metastatic and primary sites, with evidence suggesting potential long-term survival. In this case, adherence to these guidelines facilitated targeted therapy as the initial intervention, followed by surgical resection of the primary lesion post-shrinkage and radiotherapy for fibular metastasis. The patient’s favorable prognosis underscores the efficacy of this approach. It’s also important to mention that a multidisciplinary approach is essential in the management of oncological patients, with each department contributing distinct expertise to optimize diagnosis and treatment planning.

In this case, initial diagnosis was achieved through collaboration among the Orthopedics, Imaging, Pathology, and Intervention departments. Subsequent treatment planning and implementation involved coordination between Orthopedics, Thoracic Surgery, and Radiotherapy departments. Rigorous follow-up spanning 26 months has demonstrated the absence of recurrence or metastasis, affirming the success of our integrated diagnostic and therapeutic strategies.

## Conclusion

This case highlights the importance of considering metastatic disease in atypical bone lesions. A comprehensive diagnostic approach and personalized treatment with Osimertinib, followed by surgical and radiotherapeutic interventions, resulted in significant tumor reduction and sustained remission. The successful outcome underscores the efficacy of targeted therapies and multidisciplinary management. This report provides valuable insights for similar clinical scenarios, emphasizing the need for a multidisciplinary approach and tailored treatment plans to optimize patient prognosis in rare metastatic cases.

## Data Availability

The raw data supporting the conclusions of this article will be made available by the authors, without undue reservation.
